# Data Analysis in Chemistry and Bio-Medical Sciences

**DOI:** 10.3390/ijms17122105

**Published:** 2016-12-14

**Authors:** Roberto Todeschini, Alejandro Pazos, Sonia Arrasate, Humberto González-Díaz

**Affiliations:** 1 Milano Chemometrics and QSAR Research Group, Department of Earth and Environmental Sciences, University of Milano-Bicocca, 20126 Milano, Italy; roberto.todeschini@unimib.it; 2 Research Center on Information and Communication Technologies (CITIC), Institute of Biomedical Research (INIBIC), University of Coruña (UDC), Campus de Elviña s/n, 15071 A Coruña, Spain; apazos@udc.es; 3 Department of Organic Chemistry II, University of the Basque Country UPV/EHU, Sarriena w/n, 48940 Leioa, Bizkaia, Spain; sonia.arrasate@ehu.eus; 4 IKERBASQUE, Basque Foundation for Science, 48011 Bilbao, Biscay, Spain

There is an increasing necessity for multidisciplinary collaborations in molecular science between experimentalists and theoretical scientists, as well as among theoretical scientists from different fields. One of the more important forces driving this necessity is the accumulation of large amounts of data as a result of important advances in Cheminformatics and Molecular Sciences Experimental Techniques of data acquisition in general. In this context, we decided to create the MOL2NET International Conference Series on Multidisciplinary Sciences (http://sciforum.net/conferences/mol2net). The official publication platform for this conference series is SciForum, MDPI, Basel, Switzerland, and Beijing, China. The headquarters of the conference are in the Department of Organic Chemistry, University of Basque Country (UPV/EHU), Biscay, Spain.

Represented disciplines will encompass the molecular and biomedical sciences, social networks analysis, and beyond. More specifically, this conference aims to promote scientific synergies between groups of experimental molecular and biomedical scientists. Relevant fields include, but are not limited to, Chemistry, Biomedical Sciences, Neurosciences, and Nanosciences. Moreover, the conference welcomes Computational and Data Analysis experts from different areas, such as Computational Chemistry, Bioinformatics, Complex Networks Analysis, Big Data Analytics, Biostatistics, etc. The conference per se is the result of the synergy between the Department of Organic Chemistry, University of Basque Country (UPV/EHU), and IKERBASQUE, Basque Foundation for Sciences, with the Faculty of Informatics, University of Coruña (UDC).

MOL2NET-01, the first edition of this conference series, took place in December 2015 (http://sciforum.net/conference/mol2net-1). This first conference attracted more than 100 papers and 300+ authors and/or committee members representing 30+ universities of 20+ countries. Some of the world’s top universities and centers represented in the lists of committee members and/or authors were: Harvard Medical School, Boston, MA, USA; Stanford School of Medicine, Stanford, CA, USA; Virginia Commonwealth University (VCU), Richmond, VA, USA; University of Minnesota Duluth, Duluth, MN, USA; Conservatoire National des Arts et Métiers, CNAM Paris, France; University of Pennsylvania, Philadelphia, PA, USA; Miller School of Medicine, University of Miami, Coral Gables, FL, USA; EMBL-EBI European Bioinformatics Institute, Cambridge, UK; CAS Chinese Academy of Science, Beijing, China; ZJU Zhejiang University, Hanzhou, China. It is also important to highlight that the second edition of this conference, MOL2NET-02, is being held during all of 2016 and the first month of the 2017 (http://sciforum.net/conference/mol2net-02). The Special Issue of *IJMS* associated with the second edition of the conference is for 2017. This second edition keeps the modality of online participation and also started with a network of associated workshops (in-person participation) in the USA, Spain, China, and some countries of Latin America as well. 

Anyhow, we decided to assemble this Special Issue in order to strengthen and spread the outputs of MOL2NET-01. In consonance with the conference, the topic of this issue is “Data Analysis and Computational Research in Chemistry and the Bio-Medical Sciences”. The issue focuses on the development and application of different theoretical algorithms combining chemoinformatics, computational chemistry, bioinformatics, and data analysis methods. In the issue, we present a total of 18 papers with full versions of the communications presented at the conference as well as papers of other authors worldwide (direct submissions) [1,2,3,4,5,6,7,8,9,10,11,12,13,14,15,16,17,18].

In one of the papers, Sánchez and Mackenzie [1], from the University of Nebraska, Lincoln, USA (*), presented their paper entitled ”Genome-Wide Discriminatory Information Patterns of Cytosine DNA Methylation”. (*) Short reference to the affiliation of the corresponding and/or leading researcher of the manuscript, please, see the full list of affiliations, authors, and authors contributions in the final versions of the papers. Cytosine DNA methylation (CDM) is a highly abundant, heritable but reversible chemical modification to the genome. Herein, a machine learning approach was applied to analyze the accumulation of epigenetic marks in methylomes of 152 ecotypes and 85 silencing mutants of *Arabidopsis thaliana*. Results to date imply that the genome-wide distribution of CDM changes is not only part of the biological signal created by the methylation regulatory machinery, but ensures the stability of the DNA molecule, preserving the integrity of the genetic message under continuous stress from thermal fluctuations in the cell environment.

In another work, Melo et al. [2], from the University of Porto, Portugal, stated that understanding protein-protein interactions is a key challenge in biochemistry. In this work, the authors describe a more accurate methodology to predict hot spots in protein-protein interfaces from their native complex structure compared to previous published machine learning (ML) techniques.

A team lead by Syllá-Iyarreta Veitía [3], from the Conservatoire National des Arts et Métiers (CNAM), Paris, France (*), reported a quantitative structure-activity relationship (QSAR) study of the 2,2-diphenyl-l-picrylhydrazyl (DPPH) radical scavenging ability. They studied 1373 chemical compounds using DRAGON molecular descriptors and the artificial neural network (ANN) technique, a technique based on the multilayer perceptron.

Nandy and Basak [4], from the University of Minnesota (UMN), Duluth, USA (*), performed a review and discussion on the state-of-art of computer-aided drug design (CADD) techniques for the design of peptide-based vaccines. They argued about the growing incidences of new viral diseases and how increasingly frequent viral epidemics have strained therapeutic and preventive measures; the high mutability of viral genes puts additional strains on developmental efforts. In this mini-review the authors give a brief overview of some of the recent trends in computer-assisted vaccine development with emphasis on the primary selection procedures of probable peptide candidates for vaccine development.

Pastur-Romay et al. [5], from the University of Coruña (UDC), Spain (*), presented a work entitled “Deep Artificial Neural Networks and Neuromorphic Chips for Big Data Analysis: Pharmaceutical and Bioinformatics Applications”. The authors mention that over the past decade, deep artificial neural networks (DNNs) have become the state-of-the-art algorithms in ML. This was made possible by advancement in big data, deep learning and drastically increased chip-processing abilities, especially general-purpose graphical processing units. An overview of the main architectures of DNNs and their usefulness in pharmacology and bioinformatics are presented in this work. The future need for neuromorphic hardware for DNNs is also discussed, and the two most advanced chips are reviewed: the IBM TrueNorth and SpiNNaker chips. In addition, this review points out the importance of considering not only neurons, as DNNs and neuromorphic chips should also include glial cells, given the proven importance of astrocytes, a type of glial cell which contributes to information processing in the brain. The deep artificial neuron-astrocyte networks (DANAN) could overcome the difficulties in the architecture design, learning process and scalability of the current ML methods.

Aranda et al. [6] reported a quantitative structure-property relationships (QSPR) study of the soil sorption coefficient for a heterogeneous set of 643 organic non-ionic compounds. They established a conformation-independent representation of the chemical structure and analyzed 17,538 molecular descriptors calculated with the software PaDEL and EPI Suite. The present approach compares fairly well with a previously reported one which uses descriptors calculated with the software DRAGON.

De Julián-Ortiz et al. [7], from the University of Valencia, Spain (*), reported the study “Molecular Rearrangement of an Aza-Scorpiand Macrocycle Induced by pH: A Computational Study”. They stated that rearrangements and their control are a hot topic in supramolecular chemistry due to the possibilities that these phenomena open in the design of synthetic receptors and molecular machines. Macrocycle aza-scorpiands constitute an interesting system that can reorganize their spatial structure depending on pH variations or the presence of metal cations. In this study, the authors predicted relative stabilities of these conformations computationally by semi-empirical and density functional theory approximations. They also studied the reorganization from closed to open conformations which were simulated by using the Monte Carlo multiple minimum method.

Concu and Cordeiro [8], from the University of Porto, Portugal (*), presented a molecular dynamics simulation study of the selectivity of a silica polymer for ibuprofen. They commented that, in the past few years, the sol-gel polycondensation technique has been increasingly employed with great success as an alternative approach to the preparation of molecularly imprinted materials. The main aim of this study was to study, through a series of molecular dynamics (MD) simulations, the selectivity of an imprinted silica xerogel towards ibuprofen as a new template. They simulated the imprinting process occurring in a sol-gel mixture using the optimized potentials for liquid simulations-all atom (OPLS-AA) force field. To evaluate the selectivity of the polymer, they employed both the radial distribution functions, the interaction energies and the cluster analyses.

The team of authors led by Consonni and Todeschini [9], from the Università degli Studi di Milano-Bicocca, Milano, Lombardy, Italy (*), presented one study about the prediction of interactions between Cytochrome p450 and drugs. Cytochrome P450 (CYP) is the main actor in the oxidation of xenobiotics and plays a crucial role in drug safety, persistence, bioactivation, and drug-drug/food-drug interactions. This work aims to develop QSAR models to predict the drug interactions with two of the most important CYP isoforms, namely 2C9 and 3A4. The salient features of the work are (1) the thorough model validation and the applicability domain assessment; (2) the descriptor interpretation, which highlighted the crucial aspects of the P450-drug interaction; and (3) the consensus aggregation of models, which largely increased the prediction accuracy.

The team lead by Marrero-Ponce [10], from the University of San Francisco, Ecuador (*), published a paper about graph derivative indices (GDIs). This report examines the interpretation of the GDIs from three different perspectives (i.e., in structural, steric, and electronic terms). The authors claim that the individual vertex frequencies may be expressed in terms of the geometrical and electronic reactivity of the atoms and bonds, respectively. They also demonstrated that the GDIs are sensitive to progressive structural modifications in terms of: size, ramifications, electronic richness, conjugation effects, and molecular symmetry.

Besalú et al. [11], from the Institute of Computational Chemistry, University of Girona, Spain (*), presented a fast modeling of binding affinities by means of the superposing significant interaction rules (SSIR) method. They describe SSIR as a general combinatorial and symbolic procedure able to rank compounds belonging to combinatorial analogue series. The procedure generates structure-activity relationship (SAR) models and also serves as an inverse SAR tool.

The team co-directed by Ul-Haq and Barak [12], from the University of Karachi, Pakistan, and King Saud University, Saudi Arabia (*), presented a 3D-QSAR study on barbituric acid derivatives as urease inhibitors and the effect of charges on the quality of a model. The urease enzyme (EC 3.5.1.5) has been determined as a virulence factor in pathogenic microorganisms that are accountable for the development of different diseases in humans and animals. The authors presented a 3D-QSAR study based on comparative molecular field analysis (CoMFA) and comparative molecular similarity indices analysis (CoMSIA) methods. Different partial charges were calculated to examine their consequences on the predictive ability of the developed models. The analysis of obtained CoMFA and CoMSIA contour maps provided detailed insight for the promising modification of the barbituric acid derivatives with an enhanced biological activity.

Ramírez and Caballero [13], from the University of Talca, Chile (*), reported the study entitled “Is It Reliable to Use Common Molecular Docking Methods for Comparing the Binding Affinities of Enantiomer Pairs for Their Protein Target?” Molecular docking is a computational chemistry method which has become essential for the rational drug design process. In the present work the authors tested how wise is it to trust the docking energies when two complexes between a target protein and enantiomer pairs are compared. For this purpose, a ligand library composed of 141 enantiomeric pairs was used, including compounds with biological activities reported against seven protein targets. Docking results using the software Glide and AutoDock Vina were compared with the reported biological activities using a classification scheme.

Chen et al. [14], from Fujian University of Traditional Chinese Medicine, Fuzhou, China (*), presented a work focused on the structural investigation of Anthranilic acid derivatives with computational methods. In this paper, a three-level in silico approach was applied to investigate some important structural and physicochemical aspects of a series of anthranilic acid derivatives newly identified as potent partial farnesoid X receptor agonists. The authors performed both 2D- and 3D-QSAR studies based on such Anthranilic acid derivatives by a stepwise technology combined with multiple linear regression and comparative molecular field analysis. The derived contour maps from the 3D-QSAR model revealed the significant structural features (steric and electronic effects) required for improving arnesoid X receptor agonist activity.

Abdullah et al. [15], from the Forest Research Institute Malaysia and University of Malaya, Kuala Lumpur, Malaysia (*), carried out the isolation, synthesis, and QSAR study of the hyaluronidase inhibitory activity of pentacylic triterpenoids from *Prismatomeris tetrandra*. The mammalian hyaluronidase degrades hyaluronic acid by the cleavage of the β-1,4-glycosidic bond furnishing a tetrasaccharide molecule as the main product which is a highly angiogenic and potent inducer of inflammatory cytokines. The authors synthesized and assayed a series of ursolic acid analogues. The authors also reported molecular docking and QSAR analysis of their experimental results. They used several structural, topological and quantum chemical descriptors calculated with semi-empirical quantum chemical methods.

Yang et al. [16] reported a computational analysis of structure-based interactions for novel H1-antihistaminic compounds. As a chronic disorder, insomnia affects approximately 10% of the population at some time during their lives, and its treatment is often challenging. Antagonists of the H1 receptor, a protein prevalent in the human central nervous system, have been proven as effective therapeutic agents for treating insomnia. The authors studied the interaction of 129 molecules and the H1 receptor with 3D-QSAR techniques based on the CoMSIA method.

A team lead by Martín-Santamaría [17], from Centro de Investigaciones Biológicas, CIB-CSIC, Spain (*), reviewed the virtual screening methods for the discovery of toll-like receptor modulators. This review focused on the search for novel chemical entities such as modulators of Toll-like receptor (TLR) by means of virtual screening techniques. This is an emergent research field with only very recent (and successful) contributions. Identification of drug-like molecules with potential therapeutic applications for the treatment of a variety of TLR-regulated diseases has attracted considerable interest due to the clinical potential.

Lastly, a research study lead by Arrasate and González-Pinto [18], from the University of Basque Country (UPV/EHU), reported a prognostic value of affective symptoms in first-admission psychotic patients. The study focused on the use of data analysis techniques in clinical research. The authors commented that very little research has been conducted in patients with first-episode psychosis using a dimensional approach. Affective dimensional representations might be useful to predict the clinical course and treatment needs in such patients. They included 112 patients with first-episode psychosis in a longitudinal-prospective study with a five-year follow-up (*N* = 82). Logistic analyses were performed to determine the predictive factors associated with depressive, manic, activation, and dysphoric dimensions.
